# Post-COVID-19 Dysphonia: Risk, Voice Handicap, and Laryngological Findings in COVID-19 Critical Illness Survivors

**DOI:** 10.1055/s-0045-1810026

**Published:** 2025-10-16

**Authors:** Juliana Alves Souza, Carla Aparecida Cielo, Bruna Franciele da Trindade Gonçalves, Élisson Krug Oliveira, Aloma Jacobi Dalla Lana, Adriane Shmidt Pasqualoto

**Affiliations:** 1 Department of Speech Therapy and Human Communication Disorders, Federal University of Santa Maria, Santa Maria, RS, Brazil; 2 Multiprofessional Residency Program, Health Sciences Center, Federal University of Santa Maria, Santa Maria, RS, Brazil; 3 Medical Residency Program in Otorhinolaryngology, Health Sciences Center, Federal University of Santa Maria, Santa Maria, RS, Brazil; 4 Department of Physiotherapy and Human Communication Disorders, Federal University of Santa Maria, Santa Maria, RS, Brazil

**Keywords:** COVID-19, dysphonia, intensive care units, larynx, voice, voice disorders

## Abstract

**Objective:**

To investigate and relate the risk of dysphonia, voice handicap, and laryngological findings in COVID-19 critical illness survivors, stratified by sex and the need for orotracheal intubation (OTI) during hospitalization in the intensive care unit. Additionally, identify predictors of voice symptoms after COVID-19 critical illness.

**Methods:**

A cross-sectional study included 50 patients (mean age 51.70 ± 11.90 years; 26 women and 24 men) from a post-COVID-19 Rehabilitation Outpatient Clinic. Evaluations for voice symptoms and dysphonia risk were conducted using the Screening Index for Voice Disorder, vocal handicap using the Voice Handicap Index, and laryngeal health via laryngoscopic examination.

**Results:**

Dysphonia risk and voice handicap were significantly more frequent in women. There was no significant association between voice self-assessment instruments and OTI. However, voice symptoms were significantly higher in intubated women. Commonly reported voice symptoms included dry throat, throat clearing, hoarseness, and vocal fatigue. Intubation was associated with laryngeal disorders, particularly in women, with the hypopharyngeal and supraglottic regions more affected by erythema and edema. Female gender, dyspnea, and cough accounted for 51% of the variance in voice symptoms.

**Conclusions:**

COVID-19 critical illness survivors reported dry throat, throat clearing, hoarseness, and vocal fatigue even four months post-discharge. Women were at greater risk of dysphonia and voice handicaps. More than a quarter of patients presented laryngeal disorders related to OTI. Female gender and persistent symptoms of cough and dyspnea were predictors of voice symptoms. These findings enhance understanding of COVID-19's impact on the voice, highlighting the need for multidisciplinary approaches.

## Introduction

COVID-19 is a multisystem disease caused by the novel coronavirus (SARS-CoV-2), responsible for millions of deaths worldwide. 1 In the initial waves of the pandemic, approximately 20% of individuals with COVID-19 required hospitalization for pneumonia, and 5% required orotracheal intubation (OTI) and mechanical ventilation due to acute hypoxemic respiratory failure. 1 With the introduction of vaccination, the number of hospitalizations and COVID-19 related deaths has significantly decreased. Nevertheless, reinfection has contributed to additional risks, including all-cause mortality, hospitalization, and adverse health impacts, such as pulmonary, cardiovascular, neurological, and musculoskeletal sequelae. 2

COVID-19 critical illness often requires a prolonged stay in the intensive care unit (ICU), invasive ventilatory support, frequent neuromuscular blockade, and corticosteroid therapy, which may lead to more severe post-intensive care sequelae. 3 Long COVID, post-COVID-19 condition, or post-COVID-19 syndrome are characterized by the persistence or appearance of one or more symptoms that cannot be explained by an alternative diagnosis after 12 weeks of the acute phase of the illness. 1 4 It is estimated that 10–20% of individuals who have undergone an acute symptomatic phase continue to experience symptoms beyond this period, 1 with the incidence rising to 50–70% among hospitalized cases. 4 Long COVID likely has multiple, potentially overlapping causes. 4 Commonly reported symptoms include fatigue, dyspnea, cough, myalgia, headache, insomnia, and anxiety. 1 4 5

Post-COVID-19 patients may also experience conditions that can affect vocal production. 6 7 Phonation is a complex biomechanical process that is highly responsive to changes in respiratory parameters. Voice production relies on the coordination of breathing, phonation, and resonation. 8 9 10 COVID-19 mainly affects the respiratory tract, including that of the lungs, airways and musculature of the respiratory, so changes are expected not only in the voice but in the ability to produce it normally after the disease. 9 Dysphonia is a symptom that refers to a disorder in vocal quality, pitch, loudness, or vocal effort, resulting in impaired communication. 10 Dysphonia is commonly associated with upper respiratory tract infections, regardless of the causative agent (adenovirus, rhinovirus, or influenza). These viruses all induce an inflammatory response in the affected mucosa. In the case of laryngitis, the resulting edema or inflammation can restrict the vibration of the vocal folds (VF). 8 10 11 This form of dysphonia is self-limiting and typically resolves within seven to ten days, even without treatment. Other upper respiratory tract infection symptoms, including rhinitis, fever, and fatigue, are also associated with dysphonia, although voice disorders are likely to resolve spontaneously. 10 11 When dysphonia persists for more than a few weeks, further investigations should be conducted to consider other possible diagnoses, such as voice overuse, allergic laryngitis, tobacco use, head and neck cancer, medication side effects, age-related changes, intubation, and postsurgical injury, among others. 10

Post-COVID-19 dysphonia has been reported in 25–79% of patients, 8 11 12 and severity of dysphonia is associated with cough symptoms and rhinitis, 12 as well as with previous need for OTI and respiratory issues. 13 Other causes should be considered such as post-viral VF paralysis or paresis, post-viral laryngeal sensory neuropathy, vagal neuropathy, and chronic fatigue (all of which are linked to neuronal injury caused by SARS-CoV-2 - neurotropism), and psychogenic causes. 6 7 Symptoms such as dry cough, post-nasal drip, excessive sputum, throat clearing, nausea, and vomiting can lead to edema and hemorrhages in the VF, irritating the mucosa and affecting voice quality. 14 A study reported that 25% of patients hospitalized for COVID-19 and admitted to the ICU, as well as 10.3% of patients admitted to the general ward, experienced dysphonia three months after discharge. In fact, laryngological findings revealed VF paresis/paralysis in 64.3% of cases, atrophy in 28.6% of cases, and granuloma in 7.1% of cases. 15

Post-COVID-19 syndrome is considered a newly identified disease. 1 4 Nevertheless, few studies have explored these vocal symptoms and structural aspects of the larynx beyond the acute phase of the disease. 15 16 Patients' self-perception of vocal symptoms after COVID-19 including dry throat and secretion. 17 Dysphonic COVID-19 patients have been found to experience higher severity of dyspnea, sore throat pain, facial pain, and nasal obstruction compared to non-dysphonic patients. 11 A recent study revealed that dyspnea and cough symptoms may persist or worsen one year after infection in patients who were hospitalized, even with mild dysphonia. 18 Therefore, understanding the impact of SARS-CoV-2 infection on the voice is relevant in clinical practice, particularly due to the large number of post-COVID-19 syndrome patients who require rehabilitation. This knowledge is essential to ensure personalized and effective care, which can improve the quality of life of patients, as well as their work performance in cases where they need to use their voice at work.

Hence, this study sought to investigate and relate the risk of dysphonia, voice handicap, and laryngological findings in survivors of COVID-19 critical illness stratified by sex and need for orotracheal intubation during hospital admission. Furthermore, we aimed to identify predictors of voice symptoms in these patients.

## Methods

This cross-sectional study was conducted at the post-COVID-19 Rehabilitation Outpatient Clinic of a university hospital from March to November 2021. This study was approved by the Human Research Ethics Committee (process no. 4.527.287) and performed in accordance with the Declaration of Helsinki. All subjects provided written informed consent before participation. The STROBE guidelines were used to ensure the reporting of this observational study.

The inclusion criteria were patients aged 18 years or older, admitted to the ICU of this hospital with acute SARS-CoV-2 for more than two months, and who consented to participate in the research. Exclusion criteria included patients with active smoking or alcohol consumption or pre-existing medical diagnoses of neurological, cognitive, gastric, laryngeal, or voice disorders.

Initially, comprehensive patient history was taken to collect sociodemographic data, investigate persistent COVID-19 symptoms, 1 and assess habits like smoking and alcohol consumption. Medical records were reviewed to verify comorbidities and the clinical progression of COVID-19 during the hospitalization. The characteristics of these patients are detailed in Table 1 .

**Table 1:** Characterization of post-COVID-19 critical illness patients

Characteristics	Total (n = 50)		Female (n = 26)		Male (n = 24)		*p*
Age - mean ± SD	51.70	11.90	49	11.90	54.70	11.30	0.087 a
≤60 years - n (%)	36	72	21	80.77	15	62.50	0.151 d
>60 years - n (%)	14	28	5	19.23	9	37.50	
BMI - mean ± SD	34.4	6	36.1	6.41	32.5	4.97	0.035 a *
Education level - n (%)							
Elementary school	8	16	5	19.23	3	12.50	0.509 d
High school	35	70	16	61.54	19	79.17	
Higher education	7	14	5	19.23	2	8.33	
Ocupation - n (%)							
Voice professional	2	4	1	3.85	1	4.17	−
Others	48	96	25	96.15	23	95.83	
Comorbidities - n (%)							
Hypertension	32	64	17	65.38	15	62.50	0.831 d
Diabetes	13	26	5	19.23	8	33.33	0.256 d
Dyslipidemia	20	40	10	38.46	10	41.67	1 d
Heart disease	11	22	6	23.08	5	20.83	0.848 d
Lung disease	7	14	4	15.38	3	12.50	0.769 c
Kidney disease	2	4	1	3.85	1	4.17	−
Others	6	12	3	11.54	3	12.50	−
Hospitalization n (%)							
CT (% ground glass opacifications)							
< 25%	7	14	5	19.23	2	8.33	0.277 d
25-50%	12	24	8	30.77	4	16.67	
> 50%	19	38	7	26.92	12	50	
Oxygen therapy*	23	46	10	38.46	13	54.17	0.206 c
OTI	27	54	16	61.54	11	45.83	0.226 c
Days with OTI - median (IQR)	13	[8–18.8]	11	[7.75–21.3]	13	[13–17]	0.603 b
Tracheostomy	9	18	5	19.23	4	16.67	0.763 c
Days with tracheostomy - mean ± SD	33.80	17.20	28.81	16.10	40	18.70	0.365 a
Medications - n (%)							
Corticosteroids	44	88	24	92.31	20	83.33	0.744 d
Vasopressors	15	30	9	34.61	6	25	0.458 d
Neuromuscular blockers	24	48	15	57.70	9	37.50	0.095 d
Opioids	18	36	8	30.77	10	41.67	0.422 d
Prone position	11	22	5	19.23	6	25	0.738 d
Hospitalization - median (IQR) days	19	[12–30.50]	17.5	[12–25.80]	19	[12–32.30]	0.58 b
Post discharge							
Laryngeal endoscopy median (IQR) days	135	[85–263]	146	[83.5–259]	128	[85–265]	0.884 b
Long COVID symptoms - n (%)							
Dyspnea	30	60	15	57.69	15	62.50	0.729 d
Coughing	24	48	14	53.85	10	41.67	0,389 d
Fadigue	48	96	25	96.15	23	95.83	0.954 d
Myalgia	33	66	19	73.08	14	58.33	0.272 d
Sleep alteration	28	56	18	69.23	10	41.67	0.050 d *
Memory change	32	64	20	76.92	12	50.00	0.048 d *
Anxiety	12	24	20	76.92	10	41.67	0.011 d *
Headache	18	36	12	46.15	6	25.00	0.119 d
Paresthesia	23	46.9	12	46.15	11	45.83	0.982 d
Dizziness	12	24	7	26.92	5	20.83	0.614 c
Hair loss	26	52	20	76.92	6	25.00	0.001 d *
Others	17	34	9	34.62	8	33.33	0.991 d

BMI, body mass index; *oxygen therapy (mask, catheter, non-invasive ventilation); CT, computed tomography; IQR, interquartile range; n, number of patients; OTI, orotracheal intubation; SD, standard deviation.

a Student t-test for independent samples.

b Mann-Whitney U Test.

c Fisher's exact test.

d Chi-square test.

* Statistically significant Value at the level of 5% (p ≤ 0.05).

Throughout the study period, 50 consecutive patients were recruited. The sample consisted predominantly of adults who were obese, hypertensive, diabetic, and had a mean hospital stay of 19 days. More than 50% of the cases required OTI.

### Procedures

The evaluation process for the sampled patients included symptom assessment, voice handicap measurement, and endoscopic examination of the larynx.

Voice symptoms and risk of dysphonia were assessed using the Screening Index for Voice Disorder (SIVD), which consists of 12 questions related to vocal quality. Patients with a score greater than or equal to five were dysphonia risk. 19

The perception of voice handicap was quantified using the Voice Handicap Index (VHI), 20 a self-administered questionnaire consisting of 30 questions that assess different aspects of physical, emotional, and functional domains. Higher scores indicate a greater perceived disadvantage. Normative values for the scores are as follows: total = 19, functional = 7.5, emotional = 3, and organic = 10.5. 21 22 23

A 3.4-mm flexible nasolaryngoscope (Karl Storz GmbH & Co KG, Tuttlingen, Germany) was utilized for laryngeal examinations, which were performed by an otorhinolaryngologist and an experienced speech-language pathologist. The apparatus was sanitized after each examination, as per the manufacturer's recommendations. The examiner and other staff wore personal protective equipment, including face masks, protective face masks, hair protection, protective aprons, and gloves during the examination to prevent potential aerosol contamination. The integrity and appearance of anatomical structures were assessed during the structural evaluation. In the dynamic evaluation of the velopharynx, the mobility and closure of the velopharynx, as well as the mobility and symmetry of the VF, glottic closure, and VF edge during phonation, were examined.

All patients in this study entered a physical-functional rehabilitation program offered by the post-COVID-19 outpatient clinic, which emphasized aerobic training, endurance, and peripheral muscle strength. Also, those who presented voice symptoms and laryngeal disorders were referred for speech therapy and/or otorhinolaryngological evaluation and treatment. Other evaluations were carried out by specialists, and the therapeutic approach was individualized.

### Statistical Analysis

Data was analyzed using the Statistical Package for the Social Sciences software, version 20.0 (SPSS Inc., Chicago, Illinois, USA), with a significant level of 5%. The distribution of continuous variables was assessed using the Shapiro-Wilk test. Comparisons were made using the student's t-test for independent samples or the Mann-Whitney U test. The association between categorical variables was evaluated using the Chi-square or Fisher's exact test. Additionally, a multiple linear regression model (stepwise method) was used to analyze the impact of the independent variables (sex, age, OTI, long COVID-19 symptoms, and laryngological findings) on the dependent variable (SIVD and VHI scores).

The sample size was determined using the G*Power software (3.1.9.7) while considering a 25% proportion of persistent dysphonia after COVID-19 critical illness, 15 a 5% margin of error, 80% power, and an effect size of 0.2. The minimum number of patients required for the study was 46.

## Results

Table 2 presents the categorization of patients according to the SIVD and VHI scores. An analysis by sex revealed a significantly more frequent risk of dysphonia and voice handicap in women, except for the VHI emotional subscale. No significant difference was found in the vocal self-assessment instruments when stratified by the need for OTI at admission. However, women who underwent OTI demonstrated a notably higher dysphonia risk (SIVD score ≥ 5). In contrast, physical and total VHI scores were significantly elevated in women who did not receive OTI.

**Table 2:** Voice self-assessment of Post-COVID-19 critical illness patients

Instrument	General (n = 50)		Sex					OTI (n = 27)					No OTI (n = 23)					
			Female (n = 26)		Male (n = 24)		*p*	Female (n = 16)		Male (n = 11)		*p*	Female (n = 10)		Male (n = 13)		*p*	*p* (groups)
SIVD																		
>5 points - n (%)	23	46	18	69.23	5	20.83	<0.001 a *	13	81.25	2	18.18	0.007 b *	5	50	3	23.08	0.221 b	0.226 a
VHI																		
Functional score (>7.5) - n (%)	20	40	15	57.69	5	20.83	0.008 a *	9	56.25	3	27.27	0.424 b	6	60	2	15.38	0.039 b *	0.642 a
Physical score (>10.5) - n (%)	18	36	14	53.85	4	16.67	0.006 a *	9	56.25	3	27.27	0.247 b	5	50	1	7.69	0.051 b	0.254 a
Emotional score (>3) - n (%)	16	32	10	38.46	6	25	0.308 a	8	50	3	27.27	0.448 b	2	20	3	23.08	1 b	0.213 a
Total score (>19) - n (%)	21	42	16	61.54	5	20.83	0.004 a *	10	62.5	3	27.27	0.236 b	6	60	2	15.38	0.039 b *	0.474 a

n, number of patients; OTI, orotracheal intubation; SIVD, screening index for vocal disorder; VHI, vocal handicap index.

a Chi-square test.

b Fisher's exact test; Mann-Whitney U test.

* *p* <0.05.

Within the sample, 46% of participants reported experiencing voice symptoms (SIVD). Table 3 details the frequency of these symptoms, no significant difference in vocal symptoms was observed between patients who required OTI and those who did not.

**Table 3:** Post-COVID-19 critical illness patients with self-reported voice symptoms

Vocal symptoms (SIVD)	General (n = 23)		Sex					OTI (n = 15)					No OTI (n = 8)					
			Female (n = 18)		Male (n = 5)			Female (n = 13)		Male (n = 2)			Female (n = 5)		Male (n = 3)			
	n	%	n	%	n	%	*p*	n	%	n	%	*p*	n	%	n	%	*p*	*p* (groups)
Hoarseness	18	78.26	13	72.22	5	100	0.545 a	10	76.92	2	100	1	3	60	3	100.	0.464	1 a
Loss of voice	9	39.13	8	44.44	1	20	0.611	6	46.15	−	−	0.485	2	40	1	33.33	1	1
Voice failure	15	65.22	13	72.22	2	40	0.297	11	84.62	1	50	0.371	2	40	1	33.33	1	0.076
Thick voice	13	56.52	11	61.11	2	40	0.618	10	76.92	−	−	0.095	1	20	2	66.67	0.464	0.221
Clear throat	20	86.96	15	83.33	5	100	1 a	11	84.62	2	100	1	4	80	3	100	1	1 a
Dry cough	17	73.91	12	66.67	5	100	0.272 a	8	61.54	2	100	0.524	4	80	3	100	1	0.369 a
Coughing with secretion	11	47.83	8	44.44	3	60	0.640	7	53.85	1	50	1	1	20	2	66.67	0.464	0.667
Pain when talking	1	4.35	1	5.56	−	−	−	−	−	−	−	−	−	−	−	−	−	−
Pain when swallowing	8	34.78	8	44.44	−	−	0.122	6	46.15	−	−	0.488	2	40	−	−	−	0.657
Throat secretion	13	56.52	9	50	4	80	0.339	7	53.85	2	100	0.486	2	40	2	66.67	1	0.685
Dry throat	19	82.61	16	88.89	3	60	0.194	12	92.31	2	100	1	4	80	1	33.33	0.464	0.102 a
Fatigue when speaking	17	73.91	14	77.78	3	60	0.576	11	84.62	1	50	0.371	3	60	2	66.67	1	0.621 a

n, number of patients; OTI, orotracheal intubation; SIVD, screening index for vocal disorder.

a Chi-square test; Fisher's exact test.

* *p* <0.05.

**Fig. 1:**
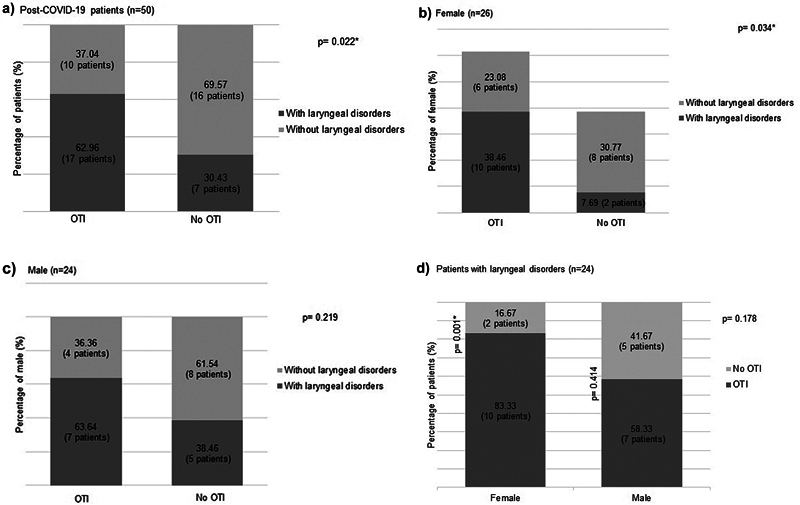
Post-COVID-19 critical illness patients distributed according to the need for orotracheal intubation (OTI), presence of laryngeal disorders, and sex. ( **a** ) Need for OTI x laryngeal disorders; ( **b** ) Need for OTI x laryngeal disorders in women; ( **c** ) Need for OTI x laryngeal disorders in men; ( **d** ) Sex x OTI need in patients with laryngeal disorders. *p < 0.05; Fisher's Exact test; Chi-square test.

Laryngeal endoscopic evaluations were conducted at a median of 135 days post-hospital discharge ( Table 1 ). Of the 50 patients evaluated, 24 (48%) exhibited laryngeal disorders during the examination, with equal prevalence among both sexes. Notably, 17 patients (70.83%) with laryngeal disorders had required OTI upon admission. Fig. 1a illustrates the significant association between the OTI and the occurrence of laryngeal disorders. Specifically, in women, a substantial association was noted between OTI and laryngeal disorders ( Fig. 1b ).

Table 4 provides the laryngological findings of post-COVID-19 patients.

**Table 4:** Laryngological findings of post-COVID-19 critical illness patients

	General (n = 24)		Sex					OTI (n = 17)					No OTI (n = 7)					
Structures			Female (n = 12)		Male (n = 12)			Female (n = 10)		Male (n = 7)			Female (n = 2)		Male (n = 5)			
	n	%	n	%	n	%	*p*	n	%	n	%	*p*	n	%	n	%	*p*	*p* (groups)
Hypopharynx																		
Edema, erythema, or pachydermia	12	50	9	75	3	25	0.014 a *	8	80	3	42.86	1	1	50.00	−	−	−	0.069
Narrowing	2	8	−	−	2	16.67	0.478	−	−	1	14.29	0.412	−	−	1	20	−	0.507
Tongue and/or epiglottis changes	4	17	1	8.33	3	25	0.590	1	10	3	42.86	0.250	−	−	−	−	−	0.283
Supraglottis																		
Edema/erythema	18	75	12	100	6	50	0.013 a *	10	100	5	71.43	−	2	100.00	1	20	−	0.306
Arching aryepiglottic folds	1	4	−	−	1	8.33	1	−	−	1	14.29	0.412	−	−	−	−	−	1
Glottis																	−	
Hypertrophy ventricular bands	1	4	1	8.33	−	−	1	1	10	−	−	1	−	−	−	−	−	1
Edema/interarytenoid thickening	5	21	1	8.33	4	33.33	0.316	1	10	3	42.86	0.250	−	−	1	20	−	1
VF mobility reduction/paralysis	5	21	3	25	2	16.67	1	3	30	2	28.57	1	−	−	−	−	−	0.272
Rounded lesions	1	4	−	−	1	8.33	1	−	−	1	14.29	0.412	−	−	−	−	−	1
Subglottis																		
Subglottic edema	3	13	−	−	3	25	0.217	−	−	3	42.86	0.062	−	−	−	−	−	0.529
Subglottic stenosis	1	4	1	8.33	−	−	1	1	10	−	−	1	−	−	−	−	−	1

n, number of patients; VF, vocal fold; OTI, orotracheal intubation.

a Chi-square test; Fisher's exact test.

* *p* <0.05.

It should be noted that there was no significant association between the patient's perception and laryngeal endoscopy findings. Of the 24 patients with laryngeal disorders in the endoscopic examination, 12 (50%) also reported vocal symptoms on the SIVD score (p = 0.942), and 8 (33%) had a high perception of the VHI total score (p = 0.598).

Table 5 presents the final regression model, which included the female sex, dyspnea, and cough as independent variables, showing a significant degree of global adjustment (R ^2^ *Adj* = 51%, p < 0.001) for the SIVD score. By analyzing the coefficients of these variables, one can observe that they are positively correlated with the SIVD score. The female sex was the variable that most influenced the SIVD score. For the VHI score, no independent variables achieved statistical significance; hence, no variable remained in the model.

**Table 5:** Influence of independent variables on the voice symptoms of post-COVID-19 critical illness patients

Dependent variable	Independent variables	R ^2^	Adjusted R ^2^	Non-standardized β	Standardized β	t	*p*
	Female sex			3.07	0.51	5.114	<0.001*
SIVD	Dyspnea	0.54	0.51	1.66	0.27	2.523	0.015*
	Coughing			2.19	0.36	3.382	0.001*

SIVD, screening index for vocal disorder.

Multiple linear regression model; *p < 0.05.

## Discussion

The underlying causes of dysphonia in COVID-19 patients appear to be multifactorial. 12 Voice symptoms in COVID-19 may arise from direct viral damage to the larynx, where SARS-CoV-2 binds to ACE2 receptors, inducing tissue inflammation. 11 Neuropathies involving the recurrent and superior laryngeal nerves and trauma or irritation of the VFs after intubation may also contribute to these symptoms. 12 Moreover, COVID-19's indirect effects on respiratory function can further complicate the vocal distress. 24

Given this context, our study sought to investigate and relate self-reported vocal assessments with laryngological findings in COVID-19 critical illness survivors stratified by sex and need for OTI during hospital admission. We observed voice symptoms and structural laryngeal changes in the disease's late phase, yet no association was found between subjective voice assessments and objective laryngeal findings. Voice symptoms were prevalent among patients regardless of intubation status, but laryngeal abnormalities were predominantly associated with intubation.

Regarding voice self-assessment, the risk of dysphonia and voice handicap was significantly more frequent in women ( Table 2 ). Evidence has shown that women are 22% more likely to exhibit post-acute SARS-CoV-2 symptoms after four weeks compared to men. 24 This may be due to women's more vigorous early innate immune response, which, while protective against the initial severity of COVID-19, might predispose them to longer-term autoimmune complications. 25 There are also anatomical and physiological issues that predispose women to voice disorders. 26 In addition to hormonal fluctuations related to perimenopause and menopause that can overlap symptoms with those of Post-COVID-19 Syndrome. 27 This is corroborated by a study, 11 which noted a significant proportion (76.6%) of female patients within the dysphonic group among mild to moderate COVID-19 survivors. Moreover, a study involving forty recovered COVID-19 patients (mean age 39.9 ± 8.8 years) reported VHI-10 scores compared to matched controls, with female patients exhibiting notably higher scores within the study cohort, 20 reinforcing our findings.

The most common voice symptoms among women included dry throat, throat clearing, vocal fatigue, voice breaks, and hoarseness. Men most frequently reported hoarseness, throat clearing, dry cough, and dry throat. No significant relationship was detected between voice symptoms and OTI necessity ( Table 3 ). Our observations regarding persistent voice symptoms are consistent with research that detected persistent dysphonia in 64.3% of female patients three months post-discharge. 15 Oxygen therapy, a common intervention during hospitalization, emerged as a predictor of self-reported vocal issues post-COVID-19. This association may be attributed to the dehydration and subsequent dryness of the vocal tract mucous membranes, 17 which could also explain the prevalent sensation of a dry throat among our patients.

Other long COVID-19 symptoms, such as cough, dyspnea, and fatigue, well-documented in the literature, 4 5 were also common in our cohort ( Table 1 ). Our regression model indicated that dyspnea, cough, and female sex could account for 51% of the variance in self-reported vocal symptoms (SIVD score) ( Table 5 ). Coughing can lead to irritation, swelling, and potential phonotrauma to the VF, 17 while dyspnea may affect the subglottic pressure essential for voice production. 8 17

Forty-eight percent of pot-COVID-19 survivors presented laryngeal disorders in the laryngoscope evaluation, significantly associated with OTI necessity ( Fig. 1a ). Regardless of the primary illness, OTI has been associated with laryngeal damage. 28 Specific clinical management practices for COVID-19, such as high-pressure ventilation, measures to prevent aerosol dispersion, prolonged intubation periods, delayed tracheotomy, and the requirement for prone positioning, may heighten the risk of enduring laryngeal injuries. 29 Moreover, the hypercoagulable and antifibrinolytic state in COVID-19 patients could lead to microvascular damage and necrosis within the tracheal and esophageal mucosa, compounded by the heightened viral replication in the tracheal epithelium, which may weaken the mucosal barrier. 30 Notably, the incidence of laryngotracheal stenosis and VF immobility post-OTI was reported to be double in COVID-19 patients compared to those without the disease. 31

Several laryngeal disorders were identified in this study, most of which were found in the hypopharyngeal and supraglottic regions. Erythema and edema were significantly more frequent in women ( Table 4 ). Considering the median time elapsed between hospital discharge and the examination (135 days), it is believed that signs of an inflammatory reaction were not detected. The late laryngological findings, such as throat clearing, dry throat, and cough, reported by most of the evaluated patients ( Table 3 ), may explain these findings. However, no significant associations were observed between patient perception and laryngological findings. Approximately two months after hospital discharge, 18.9% of the 37 post-COVID-19 patients who underwent tracheostomy and endoscopy showed laryngeal disorders (VF paralysis, subglottic stenosis, and granuloma). There was a positive correlation between the presence of laryngeal disorders and the VHI-10 score, 16 which contradicts our findings. It should be noted that the number of patients and the need for tracheostomy differed between the studies.

Regarding the laryngeal disorders observed in our study, it is worth mentioning that the patients who sought the rehabilitation outpatient clinic were obese ( Table 1 ). Laryngological findings in morbidly obese compared to eutrophic individuals showed hyperemia and edema of the VF and posterior pachyderma, related to acid laryngitis, secondary to gastroesophageal reflux. 32 Although the diagnosis of gastroesophageal reflux was an exclusion criterion in our study, we cannot rule out the possibility that this aspect influenced our findings. Obesity and diabetes are risk factors for laryngeal mucosal injury or impaired mucosal healing in patients who have been intubated for a long period due to the chronic inflammatory state associated with both conditions. 28 These comorbidities are also known severity factors for COVID-19 33 and were present in our sample, with obesity being significantly more common in women. Other factors, such as the prolonged duration of OTI and weaning from a tracheostomy, may have influenced the laryngeal findings in our study. Thus, the laryngeal findings in our study are likely related to OTI rather than COVID-19.

The pathophysiology of SARS-CoV-2 induced dysphonia may be attributed to both acute and chronic laryngeal mucosa inflammation, potentially accompanied by sensory neuropathy or vocal fold palsy. 6 7 11 17 18 Considering these factors, voice symptoms in our sample could be directly associated with COVID-19, as they were also observed in patients who were not intubated. Moreover, the regression analysis identified only female sex, dyspnea, and cough as significant predictors of the SIVD score. It should be noted that most of our samples (72%) were aged up to 60 years ( Table 1 ) and this was not influenced by SIVD and VHI scores. However, voice aging has individually variable course 34 and the incidence of voice disorders is estimated at 12 − 35% over the age of 60. 35 Therefore, studies with larger sample sizes, stratified by age, and investigating other aspects of the voice, such as acoustic and biomechanical analysis, should be conducted to confirm these findings. The relationship between persistent symptoms and voice symptoms should also be explored in future research.

This study's limitations warrant consideration. The absence of a control group of non-COVID-19 post-ICU patients limits our ability to distinguish between intubation and post-COVID-19 syndrome-related symptoms. Additionally, prior laryngoscopic evaluations were not available for comparison; however, patients with known gastric, vocal, or laryngeal issues were excluded. The influence of psychological factors on voice symptoms was not explicitly assessed, although the VHI emotional subscale provided some insight. Lastly, the single-center nature of this study, with valid results for the specific population studied, requires additional studies in different locations to confirm and generalize the findings.

## Conclusions

Survivors of COVID-19 critical illness assessed in a follow-up Rehabilitation Outpatient Clinic exhibited symptoms such as dry throat, throat clearing, hoarseness, and vocal fatigue. Notably, female patients were at greater risk of dysphonia and voice handicaps. More than a quarter of these patients presented with laryngeal disorders, which were significantly associated with OTI. Factors including female sex, dyspnea, and cough were identified as key influencers of voice symptoms. These observations underscore the extensive impact of COVID-19 critical illness on vocal health and the importance of a comprehensive, multidisciplinary approach. Furthermore, the suggestion is that otorhinolaryngologists should consider the endoscopic evaluation of the larynx in post-COVID-19 Syndrome patients who need intubation even four months after hospital discharge.
